# Prognostic impact of isolated right ventricular dysfunction in sepsis and septic shock: an 8-year historical cohort study

**DOI:** 10.1186/s13613-017-0319-9

**Published:** 2017-09-07

**Authors:** Saraschandra Vallabhajosyula, Mukesh Kumar, Govind Pandompatam, Ankit Sakhuja, Rahul Kashyap, Kianoush Kashani, Ognjen Gajic, Jeffrey B. Geske, Jacob C. Jentzer

**Affiliations:** 1 Department of Cardiovascular Medicine, Mayo Clinic, 200 First Street SW, Rochester, MN 55905 USA; 2 Division of Pulmonary and Critical Care Medicine, Department of Medicine, Mayo Clinic, 200 First Street SW, Rochester, MN 55905 USA; 3 Multidisciplinary Epidemiology and Translational Research in Intensive Care (METRIC) Laboratory, Mayo Clinic, 200 First Street SW, Rochester, 55905 MN USA; 4 Center for Clinical and Translational Science, Mayo Clinic Graduate School of Biomedical Sciences, Mayo Clinic, 200 First Street SW, Rochester, 55905 MN USA; 5 Department of Anesthesiology and Perioperative Medicine, Mayo Clinic, 200 First Street SW, Rochester, 55905 MN USA; 6 Division of Nephrology and Hypertension, Department of Medicine, Mayo Clinic, 200 First Street SW, Rochester, 55905 MN USA

**Keywords:** Sepsis, Septic shock, Right ventricle, Sepsis-related myocardial dysfunction, Mortality

## Abstract

**Background:**

Echocardiographic myocardial dysfunction is reported commonly in sepsis and septic shock, but there are limited data on sepsis-related right ventricular dysfunction. This study sought to evaluate the association of right ventricular dysfunction with clinical outcomes in patients with severe sepsis and septic shock.

**Methods:**

Historical cohort study of adult patients admitted to all intensive care units at the Mayo Clinic from January 1, 2007 through December 31, 2014 for severe sepsis and septic shock, who had an echocardiogram performed within 72 h of admission. Patients with prior heart failure, cor-pulmonale, pulmonary hypertension and valvular disease were excluded. Right ventricular dysfunction was defined by the American Society of Echocardiography criteria. Outcomes included 1-year survival, in-hospital mortality and length of stay.

**Results:**

Right ventricular dysfunction was present in 214 (55%) of 388 patients who met the inclusion criteria—isolated right ventricular dysfunction was seen in 100 (47%) and combined right and left ventricular dysfunction in 114 (53%). The baseline characteristics were similar between cohorts except for the higher mechanical ventilation use in patients with isolated right ventricular dysfunction. Echocardiographic findings demonstrated lower right ventricular and tricuspid valve velocities in patients with right ventricular dysfunction and lower left ventricular ejection fraction and increased mitral *E*/*e′* ratios in patients with combined right and left ventricular dysfunction. After adjustment for age, comorbidity, illness severity, septic shock and use of mechanical ventilation, isolated right ventricular dysfunction was independently associated with worse 1-year survival—hazard ratio 1.6 [95% confidence interval 1.2–2.1; *p* = 0.002) in patients with sepsis and septic shock.

**Conclusions:**

Isolated right ventricular dysfunction is seen commonly in sepsis and septic shock and is associated with worse long-term survival.

## Background

Sepsis-related myocardial dysfunction is frequently seen in patients with severe sepsis and septic shock [1–3]. Left ventricular (LV) systolic and diastolic dysfunction have been extensively studied in these patients and have demonstrated a variable correlation with clinical outcomes [1, 2]. In contrast, the evaluation and clinical consequences of right ventricular (RV) dysfunction in septic patients has received lesser attention [4]. RV dysfunction in sepsis is multifactorial and can be due to direct myocardial depression, hemodynamic derangements or increase in RV afterload due to hypoxemia, hypercapnia and mechanical ventilation for acute respiratory failure [5]. RV dysfunction is reported in 30–60% of all septic patients and is frequently associated with concomitant LV dysfunction [6, 7]. With the increasing use and evolution of echocardiographic methods for assessment of RV function, such as semiquantitative RV size and performance, tissue Doppler imaging (TDI) and strain imaging, there is greater evidence of RV dysfunction occurring in sepsis [3, 7].

In this study, the clinical profile and outcomes of patients with RV dysfunction in severe sepsis and septic shock were evaluated. We hypothesized that patients with RV dysfunction would have worse long-term survival and higher hospital mortality. Among patients with RV dysfunction, patients with combined RV and LV dysfunction were hypothesized to have a worse prognosis compared to those with isolated RV dysfunction.

## Methods

This historical cohort study screened all adult patients who were admitted to the intensive care units (ICU) at Mayo Clinic Rochester with severe sepsis and septic shock from January 1, 2007 through December 31, 2014. Patients with a formal, clinically indicated transthoracic echocardiogram within 72 h of ICU admission were included in this study. The characteristics of these ICU populations have been described previously [8, 9]. This study was approved by the Mayo Clinic Institutional Review Board as minimal risk to subjects and all activities were carried out in accordance with the modified Declaration of Helsinki. Patients with denial of Minnesota research authorization, known pregnancy, documented history of complex congenital heart disease, patent foramen ovale, moderate or greater valvular stenosis or regurgitation, prior heart failure, asymptomatic LV dysfunction, prior cor-pulmonale, pulmonary hypertension or recent acute coronary syndrome (<1 week) were excluded from the study.

### Data: sources, definitions and management

The 2001 American College of Chest Physicians/Society of Critical Care Medicine consensus criteria were used to define sepsis [10]. Sepsis was defined as suspicion of infection and 2/4 positive systematic inflammatory response syndrome criteria. Severe sepsis was defined as sepsis with consequent organ hypoperfusion and dysfunction as defined by lactate ≥4.0 mmol/L and/or systolic blood pressure ≤90 mmHg. Septic shock was defined as fluid-resistant hypotension (systolic blood pressure ≤90 mmHg despite ≥30 mL/kg crystalloid resuscitation) and/or use of vasopressors (norepinephrine, epinephrine, dopamine, vasopressin or phenylephrine) [11].

Patients with severe sepsis and septic shock were detected using previously validated automated search algorithms [11–13]. This algorithm has 80% sensitivity and 96% specificity for detection of severe sepsis. Demographic and clinical information was automatically abstracted from the electronic health records saved in the integrated Multidisciplinary Epidemiology and Translational Research in Intensive Care Laboratory DataMart [9, 14]. Prior acute or chronic heart failure, prior cor-pulmonale and pulmonary hypertension were evaluated using a combination of International Classification of Diseases, Clinical Modification version 9.0 diagnostic codes, pre-hospitalization echocardiogram and hemodynamic catheterization data. Laboratory, imaging and physiological parameters closest to ICU admission were abstracted. Hemodynamics, vital sign data, ventilator parameters and fluid data are collected in real time every 15 min into the DataMart and were used to coordinate data abstraction closest to the timing of echocardiography. Pre-admission echocardiography within the last 1 year was used to exclude prior ventricular dysfunction, and a combination of pre-admission echocardiogram and first hospital echocardiogram was used to exclude congenital and valvular heart disease. The severity of illness was measured using Acute Physiology and Chronic Health Evaluation III (APACHE-III) and SOFA scores. All patients with sepsis and septic shock have blood cultures and lactate levels checked, and receive 30 ml/kg intravenous fluid and antimicrobial therapy within 3 h of sepsis onset as detected by electronic search algorithm. This is a part of an ongoing quality improvement initiative in the ICUs at Mayo Clinic [11, 15].

American Society of Echocardiography (ASE) criteria were utilized for echocardiographic assessment [16]. New onset RV dysfunction was assessed using multimodality parameters as defined by the ASE criteria, i.e., specifically semiquantitative size and function, tricuspid annular plane systolic excursion (TAPSE) <16 mm by M-mode, tricuspid lateral annulus tissue Doppler systolic velocity <0.15 cm/s and RV fractional area change <35% [17]. LV dysfunction was defined as either LV systolic or diastolic dysfunction, or both. LV systolic dysfunction was defined as LV ejection fraction ≤50% [16]. LV diastolic function was classified according to standard ASE criteria, and grades II–IV were considered as diastolic dysfunction [18]. Three independent investigators (SV, MK and GP) reviewed the relevant variables and, when needed, performed manual chart reviews to ensure accuracy and fidelity of data.

The primary outcome was 1-year survival, and secondary outcomes included in-hospital mortality, ICU length of stay, ICU-free days and hospital length of stay. Mortality data were abstracted from the Mayo Clinic databases, state of Minnesota electronic death certificates and the Rochester Epidemiology Project death data system [19].

### Statistical analysis

Continuous data are presented as median (interquartile range [IQR]), and categorical data are presented as counts (percentages). Unpaired *t* test and Chi-square test were used to evaluate continuous and categorical outcomes. Odds ratio (OR) with corresponding 95% confidence intervals (CI) was used to report categorical variables in the univariate and multivariate analyses. Logistic regression and cox-proportional hazards models were used for the multivariate analysis of in-hospital mortality and 1-year survival, respectively. For the multivariate analyses, outcomes of in-hospital and 1-year mortality were analyzed using models designed from predictors with *p* < 0.10 in the univariate analysis and judgment of clinically relevant variables. Variables were assessed for collinearity prior to inclusion in the model, and only independent variables were included. The outcomes of in-hospital mortality and 1-year survival were reported using OR (95% CI) and hazard ratio (HR) (95% CI). Sensitivity analyses were performed for cohorts of patients with and without RV and/or LV dysfunction. Two-tailed *p* < 0.05 was considered statistically significant, and Bonferroni correction was used for multiple comparisons (p*k). All statistical analyses were performed with JMP version 10.0.1 (SAS Institute, Cary, NC).

## Results

**Fig. 1:**
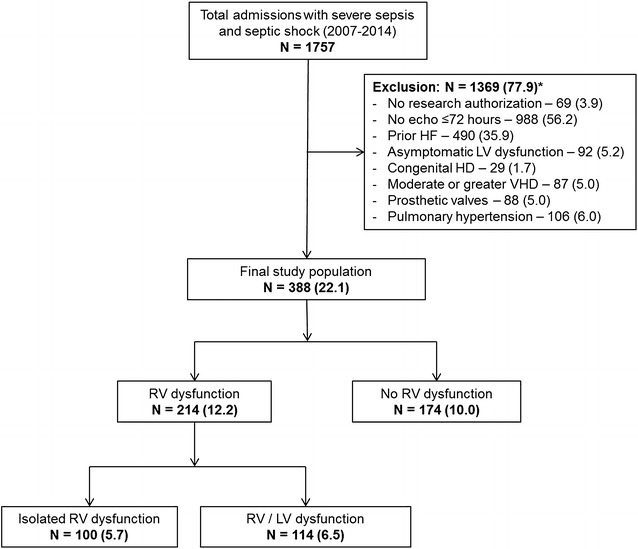
Study population. *Individual percentages are not additive due to multiplicity of exclusion criteria. *Represented as*: number (percentage). *Abbreviations*: *HD* heart disease, *HF* heart failure, *LV* left ventricular, *RV,* right ventricular, *VHD* valvular heart disease

**Fig. 2:**
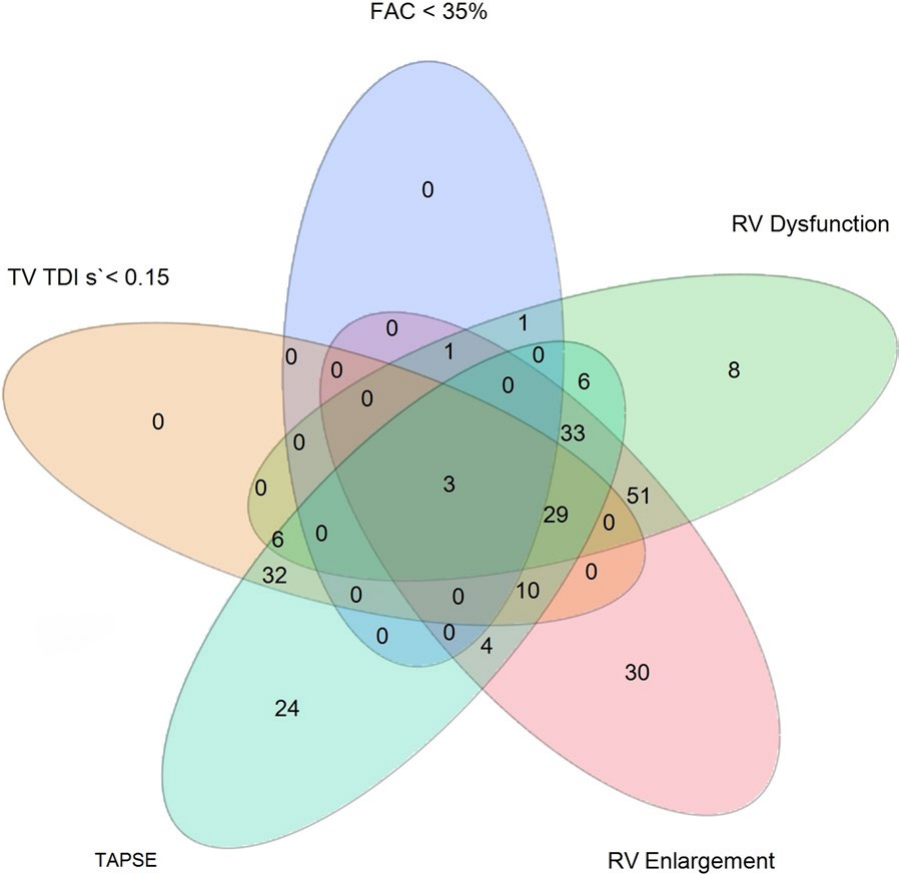
Right ventricular dysfunction using multimodality parameters. *Abbreviations*: *FAC* fractional area change, *RV* right ventricular, *s′* systolic velocity, *TAPSE* tricuspid annular plane systolic excursion, *TDI* tissue Doppler imaging, *TV* tricuspid valve

Of 1757 patients with severe sepsis and septic shock admitted to the ICUs at Mayo Clinic from 2007 to 2014, 388 (22.1%) met the eligibility criteria (Fig. 1). Using multimodality parameters, RV dysfunction was noted in 214 (55.2%) patients (Fig. 2). The patients were divided into three cohorts—isolated RV dysfunction (100; 25.8%), combined RV and LV dysfunction (114; 29.4%) and no RV dysfunction (174; 44.8%). Detailed baseline and echocardiographic parameters of the cohorts are described in Tables 1 and 2. The three cohorts differed in their severity of hypercapnia, use of mechanical ventilation and mean airway pressures during mechanical ventilation, but were comparable in all other characteristics. Patients with isolated RV dysfunction had higher associated use of invasive mechanical ventilation. RV size and function criteria were similar between isolated RV and combined RV/LV dysfunction. In keeping with the study definitions, patients with isolated RV and combined RV dysfunction had significantly lower TAPSE, and tricuspid annulus peak systolic TDI velocities than patients without RV dysfunction. Patients with combined RV/LV dysfunction had lower LV ejection fractions and higher medial *E*/*e′* ratios as compared to the other two groups (Table 2).

**Table 1:** Baseline characteristics of cohorts

Parameter	Isolated RV dysfunction (*n* = 100)	RV/LV dysfunction (*n* = 114)	No RV dysfunction (*n* = 174)	*p*
Age (years)	65.6 (55.2–77.5)	69.3 (55.3–77.4)	64.7 (53.4–74.7)	0.22
Male sex	48 (48)	59 (51.8)	91 (52.3)	0.78
Admitting location				0.13
Emergency room	53 (53)	52 (45.6)	86 (49.4)	
Hospital floors	21 (21)	28 (24.6)	55 (31.6)	
Outside transfer	26 (26)	34 (29.8)	33 (19)	
Source of sepsis				0.22
Respiratory	27 (27)	27 (23.7)	27 (15.5)	
Abdominal	2 (2)	3 (2.6)	8 (4.6)	
Genitourinary	4 (4)	6 (5.3)	18 (10.3)	
Skin/soft tissue	3 (3)	2 (1.8)	9 (5.2)	
Other/unknown	38 (38)	45 (39.5)	69 (39.7)	
Not available	26 (26)	31 (27.2)	43 (24.7)	
Body mass index (kg/m^2^)	30.8 (24.6–36.7)	28.6 (25–33.5)	28.7 (24–33.7)	0.29
Body surface area (m^2^)	2.0 (1.8–2.3)	1.9 (1.8–2.2)	1.9 (1.8–2.2)	0.27
Hypertension	35 (35)	51 (44.7)	71 (40.8)	0.35
Coronary artery disease	10 (10)	23 (20.2)	23 (13.2)	0.09
Prior myocardial infarction	7 (7)	13 (11.4)	14 (8.1)	0.48
Obstructive sleep apnea	10 (10)	19 (16.7)	22 (12.6)	0.34
Chronic lung disease	24 (24)	27 (23.7)	41 (23.6)	0.99
Charlson comorbidity index	5 (3–7)	6 (4–8)	5 (3–8)	0.40
APACHE-III score	85.5 (68.3–110)	84 (69–104)	81 (66–105)	0.54
SOFA score (day 1)	9 (7–12)	9 (7–11)	8 (5–12)	0.07
Septic shock	80 (80)	82 (71.9)	119 (68.4)	0.11
ARDS	30 (30)	36 (31.6)	49 (28.2)	0.82
Mild (n)	6	10	15	
Moderate (n)	18	18	22	
Severe (n)	6	8	12	
Acute kidney injury	62 (62)	74 (64.9)	110 (63.2)	0.91
Admission troponin-T (ng/mL)	0.06 (0.02–0.17)	0.05 (0.03–0.15)	0.06 (0.02–0.16)	0.90
Highest lactate (mmol/L)	2.8 (1.8–5.8)	3.2 (1.8–5.5)	3 (1.6–5.4)	0.86
pH	7.34 (7.26–7.39)	7.33 (7.26–7.4)	7.36 (7.29–7.42)	0.03
*p*CO_2_ (mmHg)	39 (33–45)	36 (30–44)	36 (31–42)	0.04
*Pa*O_2_/FiO_2_ ratio (mmHg)	170 (127–287)	196 (129–283)	197 (111–288)	0.87
Mechanical ventilation	67 (67)	58 (50.9)	88 (50.6)	0.03
PEEP (cm H_2_O)	7.5 (5–10)	8 (5–10)	7.5 (5–10)	0.34
PIP (cm H_2_O)	25 (18–31)	23 (17–29)	21 (14–27)	0.04
Plateau pressure (cm H_2_O)	23 (17–30)	20 (16–26)	21 (15–25)	0.17
Mean airway pressure (cm H_2_O)	14 (10–19)	14 (11–17)	13 (10–17)	0.39
Total norepinephrine (mg)	18.5 (4.7–46.8)	11.6 (3.8–33.5)	14.3 (3.8–44.3)	0.45
Crystalloid 24 h (L)	4.2 (2.4–6.8)	4.2 (2–6.2)	4.2 (2.1–7.2)	0.71

*Represented as*: total (percentage) or median (interquartile range)

*APACHE-III* Acute Physiology and Chronic Health Evaluation III therapy, *ARDS* acute respiratory distress syndrome, *FiO*_*2*_ fraction of inspired oxygen, *LV* left ventricular, *paO*_*2*_ partial pressure of arterial oxygen, *pCO*_*2*_ partial pressure of carbon dioxide, *PEEP* positive end-expiratory pressure, *PIP* peak inspiratory pressure, *RV* right ventricular, *SOFA* Sequential Organ Failure Assessment

**Table 2:** Echocardiographic parameters of cohorts*

Parameter	Isolated RV dysfunction (*n* = 100)		RV/LV dysfunction (*n* = 114)		No RV dysfunction (*n* = 174)		*p*
	*N*	Value	*N*	Value	*N*	Value	
RV enlargement	100	82 (82)	113	79 (69.9)	158	0 (0)	<0.001
RV dysfunction	100	58 (58)	114	80 (70.2)	157	0 (0)	<0.001
TR velocity (m/s)	36	2.8 (2.4–3.1)	66	2.7 (2.3–2.9)	33	2.7 (2.4–2.9)	0.22
RV systolic pressure (mmHg)	82	45 (33–58)	107	41 (33–48)	113	39 (32–46)	0.01
Estimated RA pressure (mmHg)	84	10 (5–15)	108	10 (5–14)	122	10 (5–10)	0.006
TAPSE (mm)	10	20 (13.3–23.3)	25	18 (15–19.5)	6	25.5 (21.5–28.5)	0.007
TV systolic velocity TDI (m/s)	35	0.14 (0.12–0.15)	60	0.13 (0.10–0.14)	27	0.17 (0.16–0.18)	<0.001
LV ejection fraction (%)	81	61 (56–67)	81	53 (45–61)	113	60 (55–65)	<0.001
LV end-systolic diameter (mm)	81	28 (24.5–32.5)	100	32 (28–37)	126	47 (43–51)	<0.001
LV end-diastolic diameter (mm)	87	46 (41–50)	109	47 (43–51)	111	30 (26–33)	0.17
LV mass index (g/m^2^)	73	83 (67–101.5)	100	88 (70–100)	108	90 (74–102)	0.26
LV stroke volume index (mL/m^2^)	73	42 (34.5–50)	104	37.5 (30.3–46)	116	41 (36–48)	0.009
Cardiac index (L/min/m^2^)	73	3.7 (3.1–4.5)	104	3.3 (2.8–4.1)	116	3.8 (3.2–4.4)	<0.001
Left atrial volume index (mL/m^2^)	39	21 (23–37)	63	35 (28–43)	61	33 (29–38.5)	0.16
LV peak systolic velocity (m/s)	66	0.13 (0.11–0.15)	85	0.12 (0.1–0.14)	70	0.15 (0.13–0.17)	<0.001
Mitral E velocity (m/s)	70	0.8 (0.6–1.0)	88	0.8 (0.7–1.0)	108	0.9 (0.8–1.1)	0.001
Mitral A velocity (m/s)	63	0.8 (0.6–0.9)	69	0.8 (0.6–0.9)	99	0.8 (0.7–1.0)	0.04
Mitral E/A ratio	63	1.0 (0.8–1.3)	69	1.0 (0.8–1.5)	99	1.0 (0.8–1.4)	0.44
Mitral *e*′ velocity (medial) (m/s)	68	0.08 (0.06–0.09)	91	0.07 (0.05–0.08)	105	0.07 (0.06–0.1)	0.02
Mitral *e*′ velocity (lateral) (m/s)	52	0.10 (0.08–0.13)	67	0.09 (0.08–0.10)	76	0.1 (0.08–0.12)	0.01
Mitral *E*/*e*′ ratio (medial)	65	10 (8.3–13.8)	84	12.5 (10–15)	100	12.1 (9.2–15)	0.04
Mitral *E*/*e*′ ratio (lateral)	49	7.9 (5.7–10)	61	9 (7.6–11.6)	72	9.2 (7.2–12)	0.01

*Represented as*: total (percentage) or median (interquartile range)

*LV* left ventricle, *RA,* right atrial, *RV* right ventricular, *TAPSE* tricuspid annular plane systolic excursion, *TDI* tissue Doppler imaging, *TR* tricuspid regurgitation, *TV* tricuspid valve

*Not all parameters were measured in all patients. Individual *n* for each cohort is presented in the table

### Clinical outcomes

**Fig. 3:**
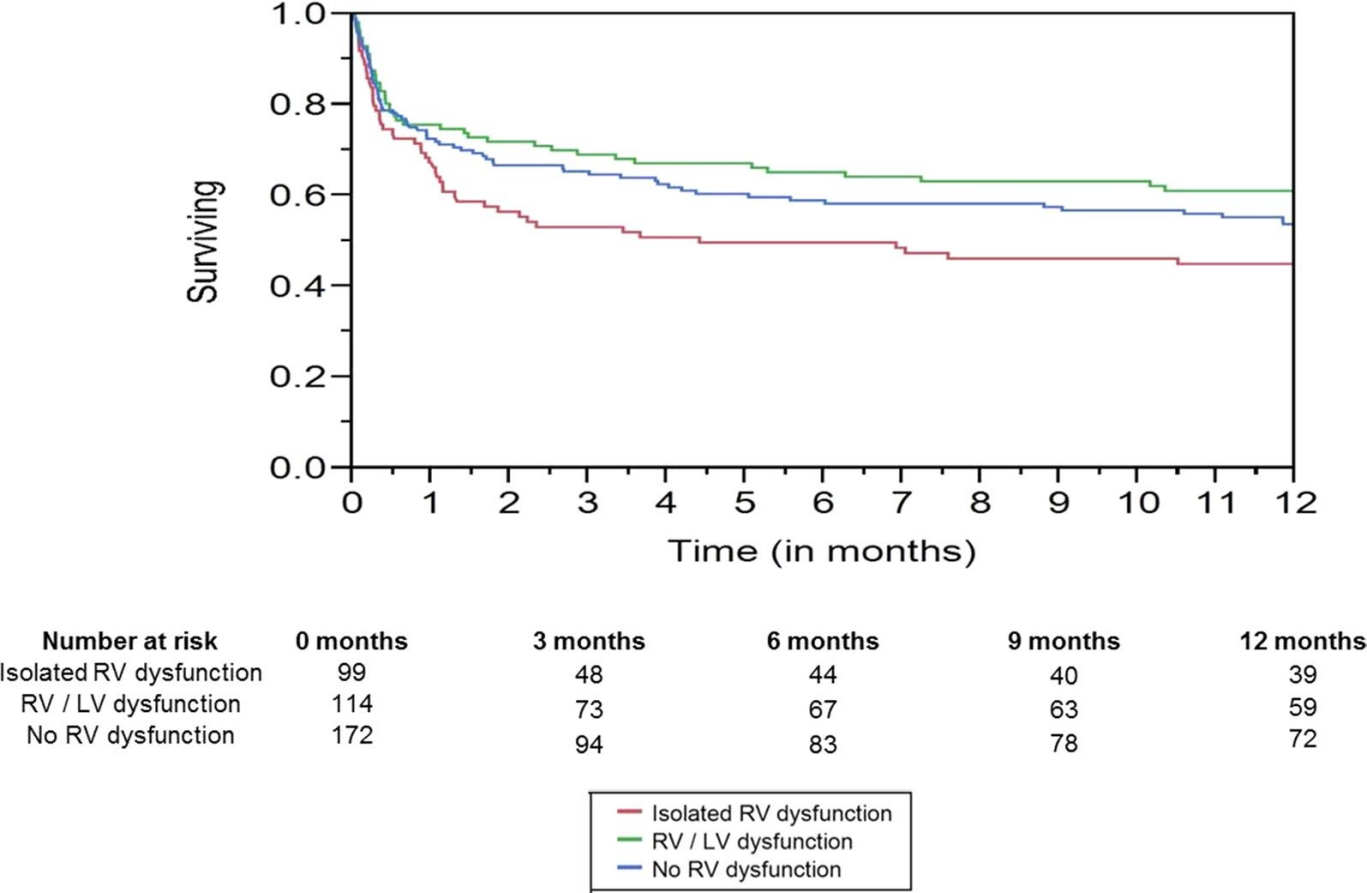
1-year survival. Log-rank test *p* = 0.003. *Abbreviations*: *LV* left ventricular, *RV* right ventricular

Unadjusted 1-year survival was significantly lower in the cohort with isolated RV dysfunction as compared to patients with no RV dysfunction or combined RV/LV dysfunction (*p* = 0.003 by log-rank test) (Fig. 3). Unadjusted in-hospital mortality (30 vs. 16.7 vs. 22.4%; *p* = 0.07), ventilator-free days (9.9 [IQR 5.2–19.1] vs. 5.6 [IQR 4.1–10.4] vs. 6.6 [IQR 4–20.7] days); *p* = 0.39), ICU length of stay (3.2 [IQR 2–6.6] vs. 3 [IQR 1.6–5.4] vs. 2.9 [IQR 1.6–6.6] days; *p* = 0.27), ICU-free days (5 [IQR 1.9–13.6] vs. 4.9 [IQR 2.7–9.2] vs. 4.9 [IQR 2.1–11] days; *p* = 0.84) and hospital length of stay (9.3 [IQR 5.8–19.4] vs. 8.5 [IQR 6–14.4] vs. 9.8 [IQR 6.1–16.6] days; *p* = 0.43) were not different between the patients with isolated RV dysfunction, combined RV/LV dysfunction and no RV dysfunction. In the admission echocardiogram, 1-year survivors had higher tricuspid regurgitant jet velocity (2.9 [IQR 2.5–3.2] vs. 2.7 [IQR 2.4–3] m/s; *p* = 0.005) and RV systolic pressure (45 [IQR 36–56] vs. 41 [IQR 33–51] mmHg; *p* = 0.002), but did not differ in semiquantitative RV size (53.1 vs. 50%; *p* = 0.53), semiquantitative RV function (45.2 vs. 47.3%; *p* = 0.66), TAPSE (17.5 [IQR 14–22.3] vs. 18 [IQR 15–21] mm; *p* = 0.90) and tricuspid annulus peak systolic TDI velocity (0.13 [IQR 0.1–0.15] vs. 0.13 [IQR 0.1–0.13] m/s; *p* = 0.76). A sensitivity analysis using visually estimated parameters (RV enlargement and RV dysfunction) only to define RV dysfunction did not demonstrate significant differences in in-hospital mortality. None of the measured echocardiographic parameters of RV function were different between hospital survivors and non-survivors.

In a cox-proportional hazards model adjusting for age, comorbidity, severity of illness, septic shock and use of mechanical ventilation, RV dysfunction was not an independent predictor of survival at 1 year in the total cohort (HR 0.9 [95% CI 0.6–1.5]; *p* = 0.83) (Table 3). Isolated RV dysfunction, however, was independently associated with worse long-term survival—HR 1.6 (95 CI 1.2–2.1), *p* = 0.002. Additional sensitivity analysis did not demonstrated combined RV/LV dysfunction to be an independent predictor of 1-year survival in the total cohort (HR 0.9 [95% CI 0.6–1.3]; *p* = 0.52).

**Table 3:** Multivariate analysis for 1-year survival with sensitivity analysis

Parameter	Univariate analysis		Multivariate analysis	
	Odds ratio (95% CI)*	*p*	Hazard ratio (95% CI)	*p*
RV dysfunction	0.9 (0.6–1.5)	0.83	0.9 (0.7–1.2)	0.40
Isolated RV dysfunction	1.5 (0.9–2.4)	0.11	1.6 (1.2–2.1)	0.002
Age (years)	1.1 (1.1–1.1)	0.007	1.0 (0.9–1.0)	1.11
Charlson comorbidity index	1.1 (1.1–1.2)	0.02	1.1 (1.1–1.1)	0.04
APACHE-III	1.1 (1.1–1.1)	<0.001	1.1 (1.1–1.1)	<0.001
Septic shock	1.9 (1.2–3.2)	0.01	1.2 (0.9–1.7)	0.24
Mechanical ventilation	1.2 (0.8–1.8)	0.58	1.1 (0.8–1.5)	0.69

*Represented as*: odds ratio (95% confidence interval) or hazard ratio (95% confidence interval)

*APACHE-III* Acute Physiology and Chronic Health Evaluation III, *CI* confidence interval

*Unit OR are presented for continuous predictors

## Discussion

RV dysfunction was noted in nearly two-thirds of patients with severe sepsis and septic shock who underwent early echocardiography in this study. Patients with RV dysfunction had higher hypercapnia and use of mechanical ventilation. When adjusted for age, comorbidity, severity of illness and use of mechanical ventilation, isolated RV dysfunction was an independent predictor of worse 1-year survival. However, presence of RV dysfunction did not impact short-term mortality and in-hospital outcomes in this study.

Prior studies on RV dysfunction in sepsis and critical illness have conflicted regarding the prognostic impact of RV dysfunction [3, 7, 20, 21]. This is likely due to heterogeneity in the timing of echocardiography, modality of echocardiography and definitions used. Consistent with this study, a recent meta-analysis did not demonstrate any correlation of semiquantitative RV size and function parameters with short-term mortality in sepsis [22]. Interestingly in our study, RV dysfunction was predictive of long-term survival. These results corroborate those by Orde et al. who demonstrated reduced RV longitudinal strain to correlate with 6-month mortality (OR 1.1 [95% CI 1.02–1.26]; *p* = 0.02) in sepsis and septic shock [21]. In this study cohort, tricuspid regurgitant jet velocity and RV systolic pressure were significantly higher in 1-year survivors; however, no difference was noted in short-term survivors. This could potentially be a reflection of the acute loading conditions in sepsis resuscitation that subsequently resolved over long-term follow-up. In addition to semi-quantitative parameters, objective parameters such as TAPSE and tricuspid annulus peak systolic TDI velocity have also been used to define RV dysfunction in patients with sepsis and septic shock. Harmankaya et al. demonstrated lower tricuspid annulus peak systolic TDI velocity (11.8 ± 4.2 vs. 13.6 ± 3.3 vs. 15.1 ± 2.1 cm/s; *p* = 0.002) in non-survivors compared to survivors and control groups, respectively [3]. The present study did not demonstrate an association between either TAPSE or tricuspid annulus peak systolic TDI velocity and mortality. TAPSE has high sensitivity in critical illness but poor specificity [23]. This could potentially be explained by the role of ventricular interdependence, the lack of control for acute right ventricular afterload that can influence the biventricular relationship and the concomitant improvement in right and left ventricular ejection fractions [4, 24]. In this current study, TAPSE showed a strong linear relationship with mitral valve lateral annulus velocity highlighting the influence of LV systolic dysfunction on TAPSE. In this study, the median LVEF in the cohort with combined RV and LV dysfunction was 53% (IQR 45–61%), representing a low incidence of isolated LV systolic dysfunction that could influence RV function.

Isolated RV dysfunction, and not biventricular dysfunction, was an independent predictor of higher long-term mortality. This was an unexpected finding that could be explained by multiple hypotheses. The RV is exquisitely sensitive to increase in afterload from lung disease, and isolated RV dysfunction could reflect cor-pulmonale from severe respiratory failure. This is consistent with the higher use of mechanical ventilation, elevated *p*CO_2_ and lower pH in this cohort from this study. However, mechanical ventilation was not a significant predictor for outcomes after adjustment for other factors in multivariate analysis. These data do not allow RV dysfunction induced or aggravated by respiratory failure to be distinguished from direct effects of sepsis on the RV itself. Alternately, prior literature has suggested that LV dysfunction is an adaptive mechanism in patients with sepsis [25, 26]. Hence, combined biventricular dysfunction might be a benign adaptive response in sepsis, whereas isolated RV dysfunction could reflect the inability of the RV to respond appropriately to stress and physiological demand [27]. Furthermore, the definitions of LV systolic dysfunction and diastolic dysfunction need further validation in the sepsis population that could influence clinical outcomes [2, 28].

This study has various limitations. Echocardiography was only performed in 44% of the population, so the prevalence of RV dysfunction among all patients with sepsis could not be evaluated. Patients without prior echocardiography and prior lung disease were included due to the low likelihood of chronic RV dysfunction; however, RV dysfunction could have been ‘unmasked’ on admission echocardiography. It is likely that patients with abnormal RV function on two-dimensional imaging underwent more detailed assessment of other RV parameters. Additionally, RV dysfunction from sepsis could not reliably be distinguished from RV dysfunction from respiratory failure due to the retrospective nature of the study. The potential influence of echocardiographic results on clinical care and outcomes could not be assessed due to the historical nature of this study. The study duration correlated with the evolution of critical care ultrasonography and changes in health care delivery at the Mayo Clinic, which conceivably could have influenced the study results. Finally, the single-region, single-institution and referral patient population of the Mayo Clinic could impact the generalizability to other populations.

Future directions for clinical research include systematically evaluating RV function in sepsis with advanced diagnostic techniques such as strain imaging that might have greater yield on homogenizing the definition of RV dysfunction. Complex heart–lung interactions, impact of mechanical ventilation and influence of volume expansion on RV function in septic patients are potential avenues for clinical and translational research. Evaluation of the pulmonary circulation using noninvasive modalities in these patients will aid in a more holistic understanding of fluid, vasopressor and ventilator management during critical illness.

## Conclusions

RV dysfunction was common in this contemporary cohort of patients with severe sepsis and septic shock that underwent echocardiography. Isolated RV dysfunction was noted to be associated with worse 1-year survival in the total cohort. These results need further validation in carefully designed prospective studies to understand the long-term significance of RV dysfunction.

## Abbreviations

APACHE: Acute Physiology and Chronic Health Evaluation
ASE: American Society of Echocardiography
CI: confidence interval
HR: hazard ratio
ICU: intensive care unit
IQR: interquartile range
LV: left ventricular
OR: odds ratio
RV: right ventricular
SOFA: Sequential Organ Failure Assessment
TAPSE: tricuspid annular plane systolic excursion
TDI: tissue Doppler imaging
